# VEGF as a Direct Functional Regulator of Photoreceptors and Contributing Factor to Diabetes-Induced Alteration of Photoreceptor Function

**DOI:** 10.3390/biom11070988

**Published:** 2021-07-05

**Authors:** Jianyan Hu, Meili Zhu, Dai Li, Qiang Wu, Yun-Zheng Le

**Affiliations:** 1 Section of Endocrinology, Diabetes and Metabolism, Department of Medicine, University of Oklahoma Health Sciences Center, Oklahoma City, OK 73104, USA; jianyanhu79@126.com (J.H.); Meili-Zhu@ouhsc.edu (M.Z.); lidai008@21cn.com (D.L.); 2 Department of Ophthalmology, Shanghai Jiao Tong University Affiliated Sixth People’s Hospital, Shanghai 200233, China; 3 School of Optometry, Hubei University of Science and Technology, Xianning 437100, China; 4 Shanghai Key Laboratory of Diabetes Mellitus, Shanghai 200233, China; 5 Department of Cell Biology, University of Oklahoma Health Sciences Center, Oklahoma City, OK 73104, USA; 6 Department of Ophthalmology, University of Oklahoma Health Sciences Center, Oklahoma City, OK 73104, USA; 7 Harold Hamm Diabetes Center, University of Oklahoma Health Sciences Center, Oklahoma City, OK 73104, USA

**Keywords:** VEGF, photoreceptors, neuronal function, ERG, diabetic retinopathy

## Abstract

Vascular endothelial growth factor (VEGF) is a major therapeutic target for blood–retina barrier (BRB) breakdown in diabetic retinopathy (DR), age-related macular degeneration (AMD), and other hypoxic retinal vascular disorders. To determine whether VEGF is a direct regulator of retinal neuronal function and its potential role in altering vision during the progression of DR, we examined the immediate impact of recombinant VEGF (rVEGF) on photoreceptor function with electroretinography in C57BL6 background wild-type (WT) and Akita spontaneous diabetic mice. Shortly after intravitreal injections, rVEGF caused a significant reduction of scotopic ERG a-wave and b-wave amplitudes and photopic ERG b-wave amplitudes in a dose-dependent manner in dark-adapted 1.5-mo-old WT mice. Compared with WT controls, 5-mo-old Akita spontaneous diabetic mice demonstrated a significant reduction in scotopic ERG a-wave and b-wave amplitudes and photopic ERG b-wave amplitudes. However, the effect of rVEGF altered photoreceptor function in WT controls was diminished in 5-mo-old Akita spontaneous diabetic mice. In conclusion, our results suggest that VEGF is a direct functional regulator of photoreceptors and VEGF up-regulation in DR is a contributing factor to diabetes-induced alteration of photoreceptor function. This information is critical to the understanding of the therapeutic effect and to the care of anti-VEGF drug-treated patients for BRB breakdown in DR, AMD, and other hypoxic retinal vascular disorders.

## 1. Introduction

Diabetic retinopathy (DR), a leading cause of blindness in the developed countries, is regarded as a diabetes-induced disorder of retinal neurons and blood–retina barriers (BRBs). Previous studies have demonstrated that the changes in retinal function and neuronal viability occur before the onset of BRB abnormalities in diabetic patients and animal models [1,2,3,4,5]. In both diabetic humans and animals, retinal function is impaired [6], as measured by changes in electroretinography (ERG) components. The viability of retinal neurons is reduced during early diabetes in streptozotocin-injected rodent models, as demonstrated by increasing neuronal apoptosis and retinal thinning, including the thinning of the inner nuclear layer (INL) and outer nuclear layer (ONL) [1,5]. These results indicate that diabetes induces the loss of neurons in all retinal layers, including photoreceptors. Diabetic patients also demonstrate a loss in green–blue color discrimination, early transition times in dark adaptation, and S-cone sensitivity [3,4]. While these observations are impactful to the field, the mechanisms of diabetes-induced alterations of visual function remain largely uninvestigated, except formulating a clear consensus that diabetes induced alterations of visual function may act as neuronal survival-dependent or -independent fashion [3,4,7].

A major accomplishment in retinal pathobiology in recent decades is the identification of vascular endothelial growth factor (VEGF or VEGF-A) as a cardinal pathogenic factor in retinal neovascularization and vascular leakage in DR and retinopathy of prematurity (ROP) and in choroidal neovascularization in age-related macular degeneration (AMD) [8,9,10,11,12], which leads to the development of anti-VEGF drugs as a major therapeutic strategy for DR, AMD, ROP, and other hypoxic ocular vascular disorders. While VEGF has been shown to act as a positive or negative regulator for neuronal function directly in the brain and peripheral neurons [13,14,15,16,17], its role as a direct functional regulator of retinal neurons has never been addressed. Potentially, elevated VEGF levels could upregulate its downstream signaling cascade and play an important role in altering neuronal function in the retina during the progression of diabetes, such as impairing visual function through alteration of visual cycle and phototransduction machinery in DR [18]. This assumption is supported by well-established observations that both VEGF receptor-1 (VEGFR1) and VEGFR2 are present in the neural retina, including photoreceptors [19,20], in which the role of VEGF as a trophic factor for retinal neurons under stress conditions has been identified [7,20,21,22,23]. To determine if VEGF plays a direct role in regulating photoreceptor function, we developed a procedure to evaluate the immediate effect of rVEGF on retinal neuronal function with ERG, based on similar approaches used in dissecting the direct role of VEGF in neuronal function in the rat hippocampus [14]. This article summarizes our work in revealing the role of VEGF in photoreceptor function using wildtype (WT) or Akita spontaneous diabetic mice [24].

## 2. Materials and Methods

### 2.1. Animals

All animal procedures were performed according to the Association for Research in Vision and Ophthalmology (ARVO) Statement for the Use of Animals in Ophthalmic and Vision Research and were approved by the Institutional Animal Care and Use Committee at the University of Oklahoma Health Sciences Center. Animals were kept in temperature controlled (25 °C) rooms with 12/12 h dark-light cycle. Akita spontaneous diabetic mice in C57Bl6 background were purchased from Jackson Laboratory (Bar Harbor, ME, USA). Genotyping of Akita mice were performed based on the instruction from the animal supplier. Heterozygous Akita mice were used in maintaining the line. Animals with blood glucose concentration over 350 mg/dL were defined as diabetic. Blood glucose concentration in diabetic animals was reconfirmed at the end-point of our experiments.

### 2.2. Electroretinography (ERG)

For measurement of retinal function with ERG, mice were dark-adapted overnight. Recombinant human VEGF (Santa Cruz Biotechnology, Dallas, TX, USA; R&D Systems, Minneapolis, MN, USA) and vehicle control solutions, which contained exactly the same PBS buffer and carrier protein (bovine serum albumin, without rVEGF) in the same volume (1 µL), were delivered to the retina intravitreally under long-wavelength illumination, according to a previous procedure [25]. Retinal function was measured with ERG using a Colordome Espion ERG recording system (Diagnosys, Lowell, MA, USA), as described previously [26,27]. Scotopic ERG was recorded using a series of flashes with increasing light intensities (from 0.002 to 400 cd·s/m^2^). Photopic ERG was performed after the mice were light-adapted in 50 cd/m^2^ background dome for 10 min. Photopic ERG was recorded with a 2000 cd·s/m^2^ flash.

### 2.3. Immunohistochemistry

To assess VEGF distribution in the mouse retina, immunohistochemistry (IHC) was performed using cryo-protected retinal sections according to a previous method [28] with the following primary antibodies: polyclonal rabbit anti-VEGF-A antibody (#ABS82 Sigma, St. Louis, MO, USA) and monoclonal rabbit anit-VEGFR2 antibody (#55B11, Cell Signaling Technology, Beverly, MA, USA) in conjunction with respective fluorescent–secondary antibodies (Thermo Fisher Scientific, Waltham, MA, USA), according to the manufacturer’s instruction. For nuclear staining, 4′,6-diamidino-2-phenylindole (DAPI) and mounting media were used according to the manufacturer’s instruction (Vector, Burlingame, CA, USA). IHC results were observed and imaged with computer-directed fluorescent microscopy.

### 2.4. Statistical Analaysis

All data required statistical analysis were expressed as mean ± SD or SEM. Statistical analysis was performed with student *t*-test or one-way ANOVA. *p*-value < 0.05 was considered statistically significant. For in vivo studies, at least eight samples were used for each experimental data point. Statistical analysis and graphic figures were made with Prism GraphPad software (San Diego, CA, USA).

## 3. Results

### 3.1. Effect of VEGF on Phtoreceptor Function

**Figure 1:**
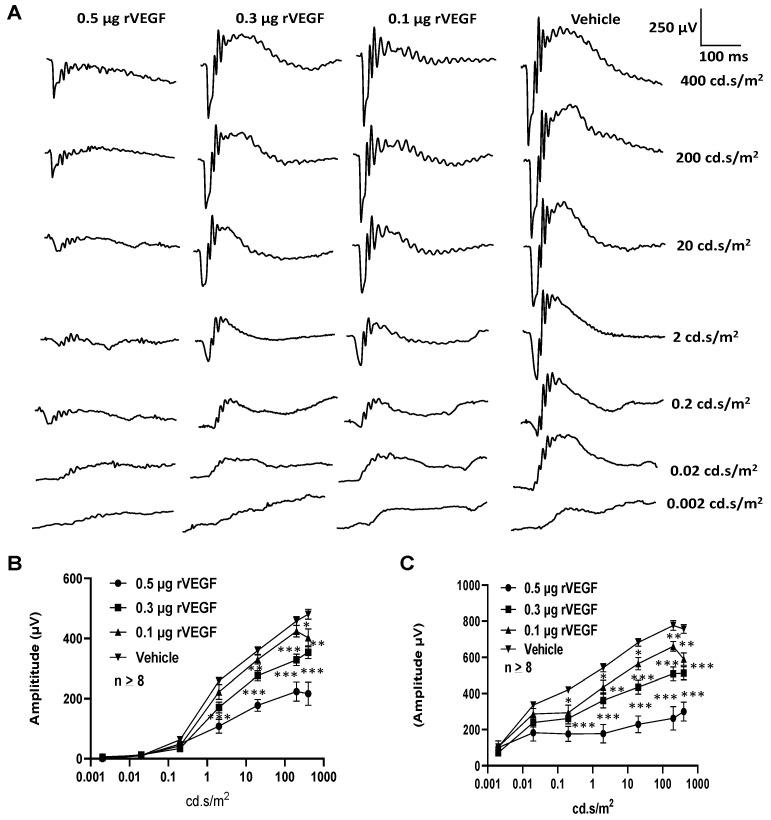
Direct effect of rVEGF on rod photoreceptor function in dark-adapted 1.5-mo-old C57BL6 mice. (**A**): Representative scotopic ERG tracers with progressive flash intensities from 0.002 to 400 cd·s/m^2^ in mice 20 min after injected intravitreally with human rVEGF (vehicle, 0.1, 0.3, or 0.5 µg/µL/eye). (**B**): Analysis of rVEGF-induced reduction of scotopic ERG a-wave amplitudes. (**C**): Analysis of rVEGF-induced reduction of scotopic ERG b-wave amplitudes. Error bar: SEM (omitted if the value was smaller than the size of the corresponding symbol in the diagram). N > 8 for all data points. ***: *p* < 0.001; **: *p* < 0.01; *: *p* < 0.05. *p*-Value < 0.05 was considered significant. rVEGF downregulated both scotopic a-wave and b-wave amplitudes in a dose-dependent manner in 1.5 mo-old WT mice.

**Figure 2:**
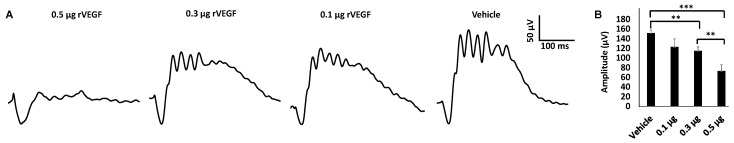
rVEGF-induced reduction of cone photoreceptor function in 1.5-mo-old C57BL6 mice in a dose-dependent manner. (**A**) Representative photopic ERG tracers with a flash intensity of 2000 cd·s/m^2^ after light adaptation at 50 cd·s/m^2^ for 10 min, which equaled to 30 min after the mice injected with human rVEGF (vehicle, 0.1, 0.3, or 0.5 µg/µL/eye) intravitreally. (**B**) analysis of rVEGF-induced reduction of photopic ERG b-wave amplitudes. Error bar: SEM. N > 8 for all data points. ***: *p* < 0.001; **: *p* < 0.01. *p*-Value < 0.05 was considered significant. rVEGF downregulated photopic b-wave amplitudes in a dose-dependent manner in 1.5 mo-old WT mice.

To determine if VEGF-A or VEGF played a directly role in regulating photoreceptor function, we intravitreally injected human rVEGF to overnight dark-adopted C57BL6 background mice (1.5-mo-old) under long-wavelength illumination and recorded ERG shortly after the injection (20 min, for sufficient diffusion of injected rVEGF). While rVEGF did not appear to alter the time to reach the trough or peak for both scotopic ERG a-wave and b-wave, rVEGF-injected animals demonstrated a dose-dependent (0.1, 0.3, or 0.5 µg rVEGF/eye) reduction of both scotopic ERG a-wave and b-wave amplitudes (Figure 1), suggesting that rVEGF was capable of downregulating rod photoreceptor function immediately after it diffused to photoreceptors and retinal neurons. The relative rVEGF-altered changes in scotopic ERG a-wave and b-wave amplitudes were reduced with time (data not shown). This observation supports that the VEGF-downregulated scotopic ERG a- and b-wave amplitudes recorded shortly after the intravitreal injection are most likely the primary responses by photoreceptors and other retinal neurons. To determine if VEGF played a directly role in regulating cone photoreceptor function, we also recorded photopic ERG in mice injected with rVEGF intravitreally. The rVEGF-injected animals demonstrated a reduction of photopic ERG b-wave amplitudes in a dose-dependent (0.1, 0.3, or 0.5 µg rVEGF/eye) manner (Figure 2), while there was no apparent change in the time-to-peak for photopic b-wave. This result suggests that VEGF may also play a similar role as a direct regulator of cone photoreceptor function.

### 3.2. Effect of VEGF on Phtoreceptor Function in Mouse Model of DR

**Figure 3:**
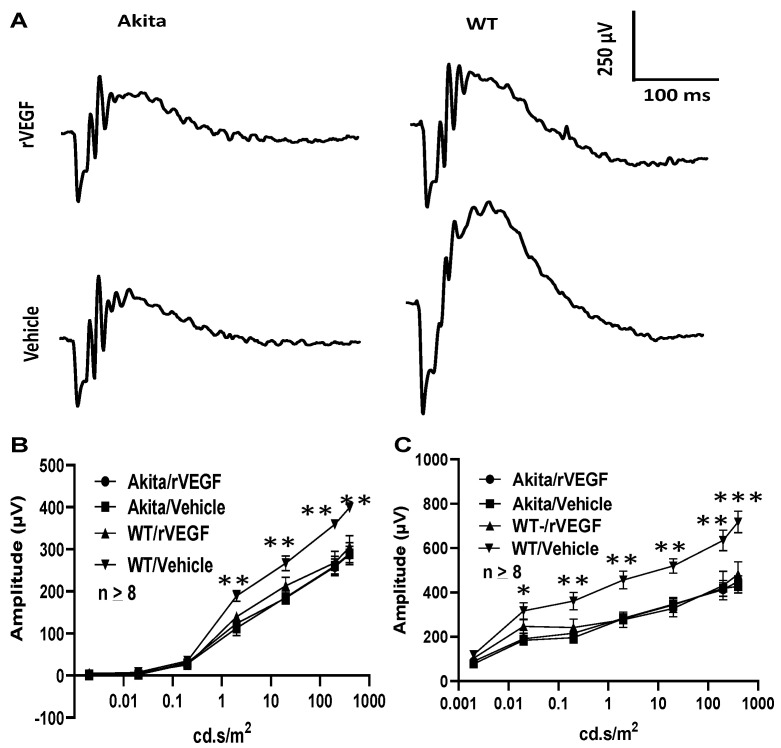
Diabetes nullified the effect of rVEGF-induced reduction of rod photoreceptor function in 5-mo-old male Akita spontaneous diabetes mice (in C57BL6 background). (**A**) Representative scotopic ERG tracers with flash intensity of 20 cd·s/m^2^ from age-matched Aktia and C57BL6 control mice 20 min after injected intravitreally with human rVEGF (vehicle or 0.3 µg/µL/eye). (**B**,**C**) Analysis of rVEGF-induced reduction of scotopic ERG a- and b-wave amplitudes. Error bar: SEM (omitted if the value was smaller than the size of the corresponding symbol in the diagram). N > 8 for all data points. ***: *p* < 0.001; **: *p* < 0.01; *: *p* < 0.05. *p*-Value < 0.05 was considered significant. Compared with WT controls, the effect of rVEGF downregulated both scotopic a-wave and b-wave amplitudes was diminished in 5-mo-old male Akita spontaneous diabetes mice.

**Figure 4:**
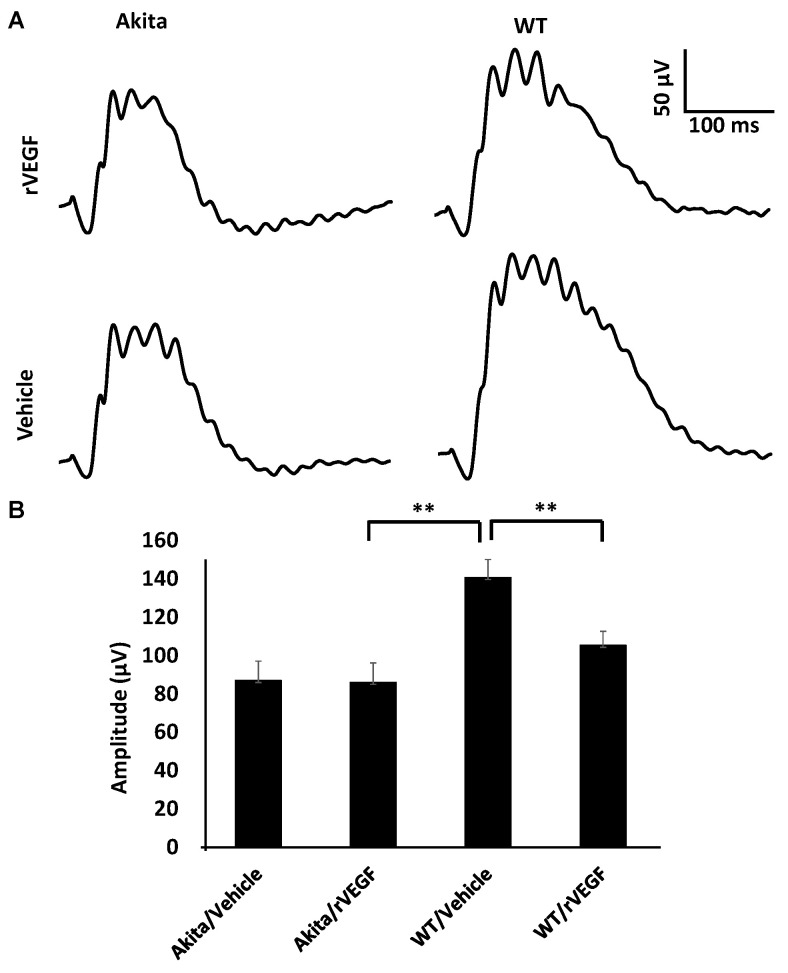
Diabetes nullified the effect of rVEGF-induced alteration of cone photoreceptor function in 5-mo-old male Akita spontaneous diabetes mice in C57BL6 background. (**A**) Representative photopic ERG tracers with a flash intensity of 2000 cd·s/m^2^ after light adaptation at 50 cd·s/m^2^ for 10 min, which equaled to 30 min after the mice injected with human rVEGF (vehicle or 0.3 µg/µL/eye) intravitreally. (**B**) Analysis of rVEGF-induced reduction of photopic ERG b-wave amplitudes. Error bar: SEM. N > 8 for all data points. **: *p* < 0.01. *p*-Value < 0.05 was considered significant. Compared with WT controls, the effect of rVEGF downregulated photopic ERG b-wave amplitudes was diminished in Akita spontaneous diabetes mice.

**Figure 5:**
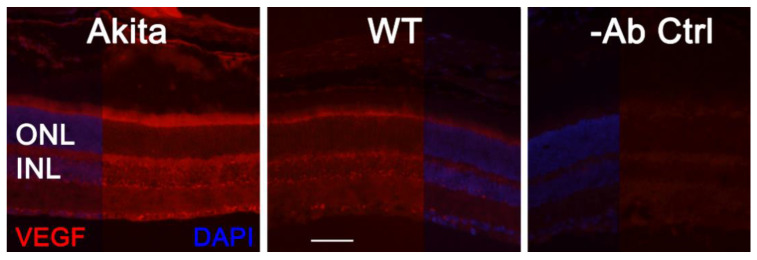
IHC detection of retinal VEGF distribution in 5-mo-old Akita spontaneous diabetes mice in (C57BL6 background) and WT controls. Scale bar: 50 µm. VEGF levels were significantly increased in the retina, particularly in the RPE, PISs, MG cell bodies, INL, ONL, and GCL. The number of VEGF-positive cells in the INL and GCL was substantially increased.

**Figure 6:**
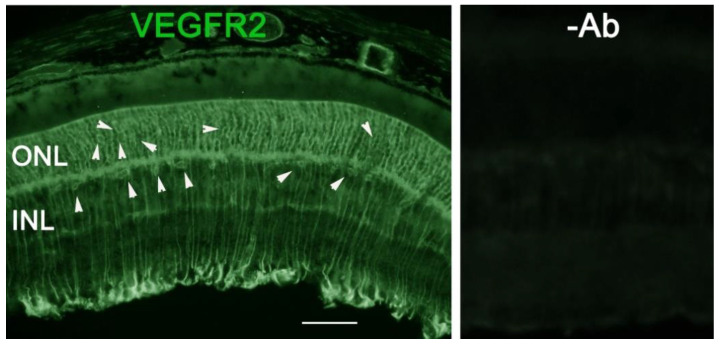
IHC detection of retinal VEGFR2 distribution in 3-mo-old C57BL6 background mice. Scale bar: 50 µm. In addition to the commonly known presence in the RPE, MG, and GCL, VEGFR2 was present in photoreceptor nuclear envelops (arrows in ONL), OPL (location of photoreceptor synaptic terminals), and INL neurons (arrows in INL) in adult WT mice.

As VEGF is gradually upregulated in the retina of diabetic mice, we asked the question whether VEGF upregulation is a contributing factor for the reduction of photoreceptor function in diabetes. We examined both scotopic and photopic ERG after intravitreal injection of rVEGF (0.3 µg/eye) or vehicle to 5-mo-old Akita spontaneous diabetic mice, which have been suggested as a suitable model for experimental DR research [24]. As expected, the Akita spontaneous diabetic mice (in C57BL6 background) demonstrated a significant loss of both scotopic ERG a- and b-wave amplitudes compared with age-matched C57BL6 WT counterparts (Figure 3). However, the effect of intravitreally delivered rVEGF on scotopic ERG a- and b-wave amplitudes was diminished in 5-mo-old Akita mice (Figure 3), which was contrary to that in WT control animals that demonstrated a similar reduction of scotopic ERG a- and b-wave amplitudes after intravitreal rVEGF injection (Figure 3), as observed in younger animals (Figure 1). Likewise, the effect of intravitreally delivered rVEGF (0.3 µg/eye) on photopic ERG b-wave amplitude was also diminished in these Akita mice (Figure 4). We then verified if retinal VEGF is gradually upregulated during the progression of diabetes in Akita mice with IHC. While the 5-mo-old WT mice demonstrated VEGF signals in the retinal pigment epithelium (RPE), photoreceptor inner segments (PIS), Müller glia (MG) cell bodies in ONL, outer plexiform (OPL), INL, ganglion cell layer (GCL), the age-matched Akita mice had a significant elevation of retinal VEGF, particularly in areas of the RPE, PIS, MG in ONL, OPL, INL, and GCL. In addition, the number of VEGF-positive cells in INL and GCL were substantially increased (Figure 5, of note, diabetic animals may be more vulnerable for detaching the RPE and sclera from photoreceptor cells due to the loss of RPE structural integrity [29]). This result suggests that the elevated level of retinal VEGF is likely a major contributing factor for diabetes-downregulated photoreceptor function in aging Akita mice. To address if VEGF receptors or/and co-receptors regulate neuronal function in photoreceptors, we also perform IHC and found that in addition to the commonly known presence in the RPE, MG, and GCL, VEGFR2, the most likely VEGF receptor for regulating neuronal function, was present in the nuclear envelops of photoreceptors (arrows in ONL), OPL (location of photoreceptor synaptic terminals), and INL neurons (arrows in INL) in 3-mo-old adult mice (Figure 6).

## 4. Discussions

The biomolecule VEGF has been identified as a direct functional regulator for a variety of neuronal activities in the brain and peripheral neurons. It serves as a depressor for synaptic transmission in hippocampus neurons and stimulus-evoked depolarization of hypoglossal motor neurons to modulate uncontrollable neurological activities, whereas its effect on sensory neurons in the brain is to promote chronic neuropathic pain [13,14,15,16,17]. These studies suggest that VEGF is capable of acting as a positive or negative regulator for neuronal functions. Since VEGF is considered as a major pathogenic factor and therapeutic target for the breakdown of BRBs in DR, AMD, and ROP, and other hypoxic retinal vascular disorders [8,9,10,11], revealing if VEGF is a direct functional regulator of retinal neurons is not only important to addressing a major knowledge gap in retinal neurobiology but is also critical to the understanding of the therapeutic effect(s) of anti-VEGF drugs. The latter is necessary to the improvement of patient care procedures during anti-VEGF therapies for these leading causes of blindness. With this in mind, we took advantage of long-wavelength illumination for intravitreal rVEGF delivery, which does not affect ERG recording for dark-adapted animals immediately after intravitreal injection. As the relative rVEGF-altered changes (percentage-wise) in scotopic ERG a-wave and b-wave amplitudes were reduced with time in our hands if we repeated the ERG hours after the initial recording (data not shown), the observed VEGF-induced reduction of scotopic ERG a- and b-waves and photopic ERG b-wave are most likely the primary responses from photoreceptors. Our method to expose rVEGF briefly to neuronal tissue is a conventional approach for dissecting specific neuronal function in the brain [14]. As rod photoreceptors comprise a majority of retinal cells in mice, the scotopic ERG results obtained with our approach is of high fidelity after allowing a short period of rVEGF diffusion. In our experimental setting, we were able to detect rVEGF-induced alteration of photoreceptor function almost immediately after intravitreal injection. In general, ERG recording with a shorter waiting time after intravitreal injection resulted in a higher variation, which is likely caused by insufficient or uneven rVEGF diffusion. The amounts of rVEGF used in our experimental setting are comparable to that in studies with transgenic VEGF expression or intraocular VEGF delivery in rodents [23,30].

To exclude the possibility that potential contaminants in the commercial rVEGF resulted in our study, we tested the human rVEGF from two independent commercial sources, as well as mouse rVEGF. All of them demonstrated a similar effect in reducing photoreceptor function in WT mice. As experiments were designed to exclude any potential VEGF-induced indirect effect on visual function and to measure the immediate and direct effect of VEGF on photoreceptor function, our results clearly support that VEGF plays a direct role in downregulating rod photoreceptor function in WT mice, a significant knowledge gap in retinal neurobiology and VEGF-related retinal pathology and diseases. However, we also recognize that (1) our work should be considered as an essential piece of evidence in the beginning of a new area and (2) the data presented were the best presentation of our work and might not address all potential issues. However, we will address them in our future work.

The data in 5-mo-old Akita spontaneous diabetic mice certainly support our experimental design. In our hands, ERG analysis suggests photoreceptor function is apparently reduced in aging Akita spontaneous diabetic mice, compared with that in WT counterparts. However, the effect of rVEGF on photoreceptor function is diminished in 5-mo-old Akita mice when there is a clear elevation of retinal VEGF. The increase of VEGF level is particularly apparent in PISs, MG in NRL, INL, and GCL (Figure 5). This result suggests that diabetes-induced VEGF upregulation nullifies the effect of intravitreally delivered rVEGF on the reduction of ERG amplitudes in normal WT mice. Therefore, it is safe to conclude that VEGF is a significant contributor to diabetes-induced reduction of photoreceptor function, at least at early stage of DR. This observation provides much needed knowledge to the understanding of altering visual function in DR and to the therapeutic effect and patient care for anti-VEGF therapies for BRB disorders.

At present, the mechanism of VEGF-induced reduction of visual function in diabetes remains elusive. Our observation that elevated VEGF presence in photoreceptor inner segments in aging diabetic mice suggests a possibility for VEGF to serve as a modulator for visual function under pathological stresses. Based on current understanding of ciliary neurotrophic factor (CNTF)- or diabetes-induced depression of visual function [18,31,32,33,34], VEGF might play a role in regulating vision through the fine tuning of phototransduction and visual cycle machineries. VEGF elevation in DR could serve as a compensatory or protective mechanism for photoreceptor machinery from uncontrollable and damaging neurological activities under diabetic and other stress conditions, which is certainly the case in CNTF-mediated depression of visual function and VEGF-mediated reduction of synaptic transmission in hippocampus neurons, as discussed above. Mechanistically, the lessons learned in the rat hippocampus [14] suggest that VEGF could exert its function through VEGF receptor-mediated signaling by regulating synaptic transmission [15]. Our observation that an enhanced VEGF level in PISs, RPE, MG, and OPL (location of photoreceptor synaptic terminals) could provide VEGF to photoreceptors and other retinal neurons for VEGF receptors- and co-receptors-mediated depressing of photoreceptor function in 5-mo-old Akita spontaneous diabetic mice (Figure 5) is certainly in agreement with this possibility. This speculation is supported by the presence of VEGF receptors and co-receptors in photoreceptors [19,20,35]. In addition to the commonly known presence in the RPE, MG, and GCL neurons, VEGFR2, was present in the nuclear envelops of photoreceptors (arrows in ONL) and INL neurons and OPL (location of photoreceptor synaptic terminals) in adult mice (Figure 6). We previously showed that the presence of VEGFR2 in photoreceptor nuclear envelops was significantly enhanced if VEGFR2 was disrupted in MG specifically [7], suggesting that this VEGFR2 IHC signal is relevant to retinal VEGFR2 signaling and is not likely derived from artifacts. More work with photoreceptor-specific VEGFR2 knockout mice will allow us to address this issue and potential mechanisms of VEGF-regulated neuronal function.

In conclusion, our work suggests that VEGF is a direct functional regulator for photoreceptors and a significant contributor to diabetes-induced alteration of photoreceptor function. This information is critical to the understanding of the therapeutic effect and to the care of anti-VEGF drug treated patients for BRB breakdown in DR, AMD, and other hypoxic retinal vascular disorders. Our findings provide new avenues to address how visual functions are affected in the progression of DR, as discussed earlier (above). We are actively exploring these potential mechanisms.

## Data Availability

All data presented in this work are included in the article.
